# Rs1800625 in the receptor for advanced glycation end products gene predisposes to sepsis and multiple organ dysfunction syndrome in patients with major trauma

**DOI:** 10.1186/s13054-014-0727-2

**Published:** 2015-01-09

**Authors:** Ling Zeng, Juan Du, Wei Gu, An-qiang Zhang, Hai-yan Wang, Da-lin Wen, Lin Qiu, Xue-tao Yang, Jian-hui Sun, Mao Zhang, Jiang Hao, Jian-xin Jiang

**Affiliations:** State Key Laboratory of Trauma, Burns and Combined Injury, Institute of Surgery Research, Daping Hospital, Third Military Medical University, 10 Changjiang Road, Yuzhong District, Daping, Chongqing, 400042 China; Biochemistry and Molecular Biology Laboratory of Experiment Teaching Center, Chongqing Medical University, Fengyu Road, Chongqing, 401331 China; Department of Emergency Medical Center, the Second Affiliated Hospital, Zhejiang University, 88 Jiefang Road, Zhejiang, 310009 China; Kunming General Hospital, Chengdu Military of PLA, 212 Grand View Road, Kunming, Yunnan 650032 China

## Abstract

**Introduction:**

The receptor for advanced glycation end products (RAGE) is a transmembrane receptor of the immunoglobulin superfamily, it plays pivotal roles in the pathogenesis of sepsis in several ways. Our previous study showed that rs1800625 (−429T/C) revealed a strong clinical relevance with sepsis morbidity rate and multiple organ dysfunction syndrome (MODS) in patients with major trauma. In this study, we enlarged the sample size, added two validation populations and examined the expression of RAGE on the surface of peripheral leukocytes to *ex vivo* lipopolysaccharide (LPS) stimulation in subjects with different genotypes.

**Methods:**

Rs1800625 was genotyped using pyrosequencing in 837 Chinese Han patients with major trauma in Chongqing. We then validated the clinical relevance in 340 Zhejiang and 347 Yunnan patients. The expression of RAGE on the surface of peripheral blood mononuclear cells was measured by flow cytometric analysis.

**Results:**

The results indicated that rs1800625 was significantly associated with sepsis morbidity rate and MODS in patients with major trauma in the Chongqing, Zhejiang and Yunnan districts. Patients with CC genotype had lower sepsis morbidity rate and MODS after major trauma. Furthermore, patients with CC genotype had significantly higher RAGE expression (*P* = 0.009).

**Conclusions:**

The rs1800625 polymorphism is a functional single nucleotide polymorphism and confers host susceptibility to sepsis and MODS in patients with major trauma.

## Introduction

The traditional view of trauma as ‘accidents’, or random events, has resulted in the historical neglect of this area of public health. The most recent estimates show that trauma is among the leading causes of death and disability in the world. In 1998, about 5.8 million people (97.9 per 100,000 population) died of trauma worldwide, and trauma caused 16% of the global burden of disease [1]. Trauma affects mostly young people, often causing death or long-term disability. With great improvements in the emergency care system, the majority of severe trauma patients survived traumatic injury. However, many still suffer from complications after admission, leading to in-hospital death. Sepsis and multiple organ dysfunction syndrome (MODS) are common and severe complications in trauma patients [2]. Therefore, preventing sepsis and MODS is critical to the treatment of patients who survive major trauma. Because of this, establishing an early diagnosis of sepsis and MODS is an essential prerequisite.

The receptor for advanced glycation end products (RAGE) is a transmembrane receptor of the immunoglobulin superfamily; it has been recognized as a multi-ligand receptor. RAGE plays pivotal roles in innate immune responses as a pattern-recognition receptor (PRR) in sensing both ‘pathogen-associated molecular patterns’ (PAMPs) and endogenous damage-associated molecular patterns (DAMPs). Ligands that have been found to be recognized by RAGE include advanced glycation end products (AGEs), amyloid β-peptide, DNA-binding protein high-mobility group box-1 (HMGBl) and S100/calgranulins [3-7]. RAGE has been suggested to be involved in the pathogenesis of sepsis in several ways. The cellular effects resulting from the activation of RAGE by the above-mentioned ligands are mediated by multiple intracellular signaling pathways, including nuclear factor-κB (NF-κB), leading to the transcription of proinflammatory factors [8]. RAGE deficiency improved survival in a model of abdominal polymicrobial sepsis induced by cecal ligation and puncture (CLP) [9].

Our previous study shows that rs1800625 reveals a strong clinical relevance, showing lower sepsis morbidity rates and MODS scores in patients with the variant C allele [10]. Using a reporter gene assay system, we investigated the effect of rs1800625 on RAGE promoter activity. The results suggested that T to C variation at position −429 could significantly reduce the transcriptional activity of RAGE promoter. Our further study showed that rs1800625 was significantly associated with lower responsiveness of peripheral blood leukocytes in response to lipopolysaccharide (LPS) stimulation, showing much lower levels of tumor necrosis factor alpha (TNFα) in patients carrying the C allele. Thus, we enlarged the sample size and added two validation populations to further investigate the clinical relevance of -429 T/C with the development of sepsis and MODS in patients with major trauma. Furthermore, to examine whether there is a functional linkage between rs1800625 and membrane-bound RAGE protein expression, we examined the expression of RAGE on the surface of peripheral leukocytes to *ex vivo* LPS stimulation in subjects with different genotypes.

## Materials and methods

### Study populations and clinical evaluation

Three independent patient cohorts were recruited for this study, Chongqing (n = 837), Zhejiang (n = 340) and Yunnan (n = 367). They were admitted to the Department of Trauma Surgery in the Daping Hospital and the Chongqing Emergency Medical Center between 1 January 2005 and 1 October 2013, and to the Department of Trauma and Emergency in the Second Affiliated Hospital, Zhejiang University between 1 January 2008 and 1 October 2013 and the Department of Trauma and Emergency in the General Hospital of Kunming between 1 January 2007 and 1 October 2013 in Yunnan province. They were enrolled in the study if they met the following inclusion criteria: (1) between 18 and 65 years of age, (2) expected Injury Severity Score (ISS) greater than 16 combined with the presence of at least one life-threatening injury and at least one additional severe injury in another part of the body. Patients were not eligible if they had penetrating injuries or preexisting cardiovascular, respiratory, renal, hepatic, hematologic or immunologic diseases. ISSs were determined by independent evaluators in accordance with the abbreviated injury scale developed in 2005 [11]. All patients requiring surgical intervention received standard surgical care and postoperative intensive care unit (ICU) treatment. The patients from Chongqing district constituted the inception cohort, which was used to screen the single nucleotide polymorphisms (SNPs) with possible clinical relevance. The patients from Yunnan and Zhejiang districts constituted the validation cohorts. The protocol was approved by the Ethical and Protocol Review Committee of the Third Military Medical University, and informed consent was obtained from the patients and the patients’ next of kin. Patient confidentiality was preserved according to the guidelines for studies of human subjects.

The diagnosis of sepsis met the criteria recommended by the American College of Chest Physicians and Society of Critical Care Medicine Consensus Conference [12]. Infection was defined as a clinically obvious source or positive bacterial cultures. Pneumonia was diagnosed when a predominant organism was isolated from appropriately obtained sputum cultures in the setting of purulent sputum production and/or a new or changing pulmonary infiltrate on patients’ chest X-ray film. Bloodstream infections were diagnosed based on isolation of a predominant organism from blood cultures obtained under sterile conditions. Criteria for urinary tract infections included the presence of clinical symptoms and >10 white blood cells/high-power field on microscopic examination or isolation of >10^5^ organisms/ml of urine or >10^4^ organisms. Criteria for catheter-related infections included isolation of >15 colony-forming units from catheter tips cultured only when infection was suspected. Wound infection was identified by drainage of purulent material from the wound. Daily physiologic and laboratory data were collected during the hospital stay and clinical events were recorded thereafter until death or discharge from the hospital [13,14]. Multiple organ dysfunction scores were calculated as the sum of the simultaneously obtained individual organ scores on each hospital day [15]. MODS scores and the presence of sepsis were determined by individuals who did not know the patients’ genotypes.

### Genotyping

Genomic DNA was isolated from whole blood using Wizard genomic DNA purification kit (Promega, Madison, WI, USA). Pyrosequencing was used for genotyping of rs1800625 according to our previous reports [13,14]. The PCR primers and the annealing temperature were shown in Table 1. Genotyping was performed in a blinded fashion without knowledge of the patients’ clinical data, and 10% of the samples were further confirmed by direct sequencing.

**Table 1:** **Primers and PCR conditions**

**SNP**	**Primers for PCR**	**Sequencing primer**	**SNP sequence**	**Annealing temperature**
rs1800625	F^: *^bio-TCTTTTTTCCCTGGGTTTAGTTGA	GAGAGAAACCTGTTTGG	AA/GCTTC	60°C
	R: ATAGGGTTCAGGCCAGACTGTTGT			

^*^biotin labeling.

### Flow cytometric analysis

The expression of RAGE on the surface of peripheral blood mononuclear cells was measured by flow cytometric analysis. The whole blood samples collected from 42 healthy volunteers were mixed at a dilution ratio of 1:1 (vol/vol) using RPMI 1640 culture medium, and incubated with 100 ng/ml of *Escherichia coli* LPS (O26:B6) at a temperature of 37°C for 4 hours. LPS-activated peripheral blood mononuclear cell samples at a density of 1 × 10^6^ cells/ml were stained with anti-human RAGE monoclonal antibody (Abcam, Cambridge, UK). Then cells were stained with fluorescein isothiocyanate (FITC)-conjugated donkey anti-mouse immunoglobulin G (IgG) (Life Technology, Carlsbad, CA, USA). After washing, the proportion of RAGE-positive cells was determined using flow cytometer. The RAGE expression on monocytes was presented as mean fluorescence intensity (MFI).

### Statistical analysis

Sample size was calculated using online Power and Sample Size Program software [16]. The desired power of our study was set at 80% with a significance level of 0.05 in a two-sided test. We chose the log-additive inheritance model, which is the most suitable for polygenic diseases.

Allele frequency was determined by gene counting. Genotype distribution was tested for departure from the Hardy-Weinberg equilibrium (HWE) using χ^2^ analyses. The extent of pairwise linkage disequilibrium between polymorphisms was determined by the Haploview (version 4.0) software. The association between polymorphisms and MOD scores was performed using analysis of one-way analysis of variance (ANOVA) testing with age, sex ratio, and injury severity to adjust for possible confounding effects. Three genetic models (dominant, recessive and allele-dose effects) were used. The association of genotypes with sepsis morbidity rate was determined by χ^2^ analysis. Odds ratios with 95% confidence intervals were calculated by multiple logistic regression analyses to estimate the relative risk of sepsis. Age, sex ratio, and injury severity were used as covariances of multiple logistic regression. All statistical analysis was carried out using SPSS version 13.0 (SPSS Inc, Chicago, IL, USA) [13,14].

## Results

### Study population and clinical evaluation

Three independent patient cohorts were recruited for this study, Chongqing (n = 837), Zhejiang (n = 340) and Yunnan (n = 367). All patients survived at least 48 hours after admission. Baseline data of the patients are shown in Table 2. Patients were mostly young in age (mean age 41.1 ± 13.4, 42.5 ± 12.0 and 37.6 ± 12.5), and had sustained severe injuries (mean ISS 22.3 ± 9.4, 21.7 ± 7.7, and 21.3 ± 9.2). Sepsis morbidity rate was 41.3%, 37.9% and 35.4% in Chongqing, Zhejiang and Yunnan cohorts. Organ dysfunction occurred in 51.4%, 56.2%, and 57.5% of patients in the Chongqing, Yunnan, and Zhejiang cohorts, respectively, among which 162 (19.4%), 79 (23.2%), and 86 (23.4%) patients exhibited dysfunction in two or more organs. Median time point for sepsis occurrence in the whole study cohort was 6 days (interquartile range 5.0 to 8.5 days). Median time point for organ dysfunction occurrence in the whole study cohort was 7 days (interquartile range 5.0 to 8.5 days).

**Table 2:** **Overall clinical characteristics of patients with major trauma**

	**Screening cohort**	**Validation cohorts**	
	**Chongqing (N = 837)**	**Zhejiang (N = 340)**	**Yunnan (N = 367)**
Age (yrs)	41.1 ± 13.4 (18-65)	42.5 ± 12.0 (19-65)	37.6 ± 12.5 (18-65)
Male/female, n	672/165	266/74	282/85
Injured body regions, n (%)			
Head, n	458 (54.7)	248 (72.9)	225 (61.3)
Thorax, n	513 (61.2)	209 (61.5)	186 (50.7)
Abdomen, n	389 (46.4)	118 (34.7)	105 (28.6)
Extremities, n	425 (50.8)	179 (52.6)	196 (53.4)
Number of regions injured, n (%)			
One, n	435 (52.0)	146 (42.9)	157 (42.8)
Two, n	267 (31.9)	129 (37.9)	131 (35.7)
Three or above, n	135 (16.1)	65 (19.1)	79 (21.5)
ISS	22.3 ± 9.4	21.7 ± 7.7	21.3 ± 9.2
≥16, <25, n (%)	510 (60.9)	197 (57.9)	228 (62.1)
≥25, n (%)	327 (39.1)	143 (42.1)	139 (37.9)
Organ dysfunction, n (%)			
One, n	268 (32.2)	112 (32.9)	125 (34.1)
Two, n	121 (14.5)	56 (16.5)	51 (13.9)
Three or above, n	41 (4.9)	23 (6.8)	35 (9.5)
Sepsis, n (%)	346 (41.3)	129 (37.9)	130 (35.4)
Source of infection, %			
Respiratory tract infection	41.6	39.7	43.2
Primary bloodstream infection	21.5	24.1	19.9
Urinary tract infection	16.2	11.2	12.1
Catheter-associated infection	10.9	9.1	9.3
Wound infection	7.4	9.1	10.1
Others^*^	2.4	6.8	5.4
Pathogens, % (positive blood cultures)			
Gram-negative	22.3	25.7	29.5
Gram-positive	17.6	13.2	12.8
Fungi	2.8	3.5	3.6
Mixed Gram-negative and -positive	5.9	7.5	8.5
Negative blood cultures	54.0	55.0	57.0

ISS, Injury Severity Score.

### Clinical relevance of rs1800625 in trauma patients from Chongqing district

First, 837 Chinese Han patients with major trauma from Chongqing district were used to investigate the clinical relevance of rs1800625. The minor allele frequency (MAF) of rs1800625 was 14.2% in Chongqing districts (Table 3). The genotype distribution was in agreement with the HWE (*P* >0.05, Table 3).

**Table 3:** **Distribution of rs1800625 of the RAGE gene among trauma patients in the three cohorts**

	**N**			**Genotypes**			**Hardy-Weinberg** **equilibrium**
		**Patients**	**Databank**	**Wild**	**Heterozygous**	**Variant**	
**Chongqing**	837	14.2	12.2	620	195	22	0.16
**Zhejiang**	340	10.7		271	65	4	0.96
**Yunnan**	367	14.9		269	86	12	0.12

As shown in Table 4, there were no significant differences in age, gender ratio and ISS among patients stratified according to the different genotypes of rs1800625. The patients carrying T allele of rs1800625 revealed significantly increased risk of sepsis and higher MODS scores when compared to those carrying C allele (*P* = 0.002 for sepsis morbidity rate and *P* = 0.001 for MODS scores in case of dominant effect, *P* = 0.032 for MODS scores in case of recessive effect). Data from regression analyses further indicated that the association of this polymorphism was in significant allele-dose effect with sepsis morbidity rate (odds ratio (OR) = 0.378, 95% confidence interval (CI): 0.254 to 0.893, *P* = 0.017).

**Table 4:** **Clinical relevance of rs1800625 among trauma patients in the three cohorts**

	**Genotypes**	**N**	**Age (yr)**	**Sex (M/F)**	**ISS**	**Sepsis, n/%**	**MODS score**
**Chongqing**	TT	620	40.8 ± 13.1	494/126	22.6 ± 9.3	275 (46.8%)	6.9 ± 2.2
	TC	195	41.9 ± 14.5	157/38	21.5 ± 9.7	65 (33.3%)	6.0 ± 2.0
	CC	22	41.0 ± 11.4	21/1	20.0 ± 9.4	6 (27.3%)	5.2 ± 1.7
						a1	a2, b1
**Zhejiang**	TT	271	42.3 ± 12.7	212/59	21.7 ± 7.7	113 (41.7%)	7.5 ± 2.5
	TC	65	42.8 ± 16.2	50/15	21.9 ± 7.6	15 (23.1%)	6.8 ± 2.6
	CC	4	45.8 ± 19.8	4/0	18.8 ± 6.5	1 (25.0%)	5.9 ± 3.1
						a3	a4
**Yunnan**	TT	269	37.4 ± 12.5	208/61	21.3 ± 9.0	103 (38.3%)	7.2 ± 2.0
	TC	86	38.7 ± 12.0	64/22	20.8 ± 9.8	24 (27.9%)	6.3 ± 2.1
	CC	12	33.8 ± 14.4	10/2	24.8 ± 8.7	3 (25.0%)	5.8 ± 2.6
						a5	a6

a: dominant effect (variant homozygotes + heterozygotes vs. wild homozygotes) as analyzed by ANCOVA, ^a1^ *P* = 0.002, ^a2^ *P* = 0.001, ^a3^ *P* = 0.003, ^a4^ *P* = 0.032, ^a5^ *P* = 0.047, ^a3^ *P* = 0.016, ^a4^ *P* = 0.042, ^a5^ *P* = 0.012, ^a6^ *P* = 0.026; b: recessive effect (variant homozygotes vs. heterozygotes + wild homozygotes) as analyzed by ANCOVA, ^b1^ *P* = 0.032. ISS. Injury Severity Score; MODS, multiple organ dysfunction syndrome.

### Validation of the clinical relevance of rs1800625 in Zhejiang and Yunnan trauma patients

We further validate the results in two additional distinct cohorts (Zhejiang and Yunnan populations). Zhejiang (N = 340) and Yunnan (N = 347) provinces are located in eastern and southern China. The characteristics of trauma patients in these two validation cohorts are presented in Table 1. The overall MAF of rs1800625 in Zhejiang and Yunnan populations are similar to those in Chongqing. The genotype distribution is in agreement with the HWE. As shown in Table 4, rs1800625 was also shown to be strongly associated with risk of the development of posttraumatic sepsis in both independent validation cohorts. The patients carrying the variant T allele had significantly higher sepsis morbidity rate than those carrying the wild C allele (*P* = 0.016 for sepsis morbidity rate and *P* = 0.042 for MODS scores in case of dominant effect in Yunnan cohorts. *P* = 0.016 for sepsis morbidity rate and *P* = 0.042 for MODS scores in case of dominant effect in Zhejiang cohorts. *P* = 0.012 for sepsis morbidity rate and *P* = 0.026 for MODS scores in case of dominant effect in Yunnan cohorts).

### Association of rs1800625 with RAGE protein expression

**Figure 1:**
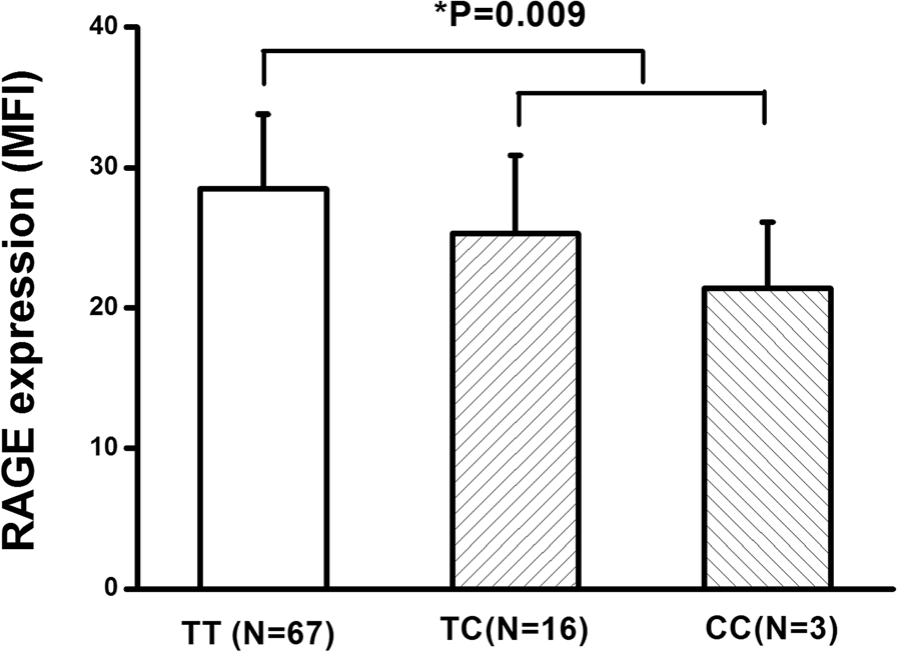
**Association of the rs1800625 with LPS-induced expression of RAGE protein.** The whole blood samples were collected from 42 healthy volunteers (TT: N = 32, TC: N = 8, CC: N = 2). The RAGE expression on the peripheral leukocytes was assayed using flow cytometry and was presented as MFI. One-way ANOVA was used to assess statistical significance. *P* = 0.009 for dominant association (CC + TC vs. TT). ANOVA, analysis of variance; LPS lipopolysaccharide; RAGE, the receptor for advanced glycation end products.

To examine whether there is a functional linkage between rs1800625 and membrane-bound RAGE protein expression, we examined the expression of RAGE on the surface of peripheral leukocytes to *ex vivo* LPS stimulation in subjects with different genotypes. The RAGE expression was shown to be well associated with rs1800625 polymorphism, showing a significant difference in case of dominant effects (*P* = 0.009) (genotype MFI: CC, 28.5 ± 5.3; TC, 25.3 ± 5.6; CC, 21.4 ± 4.7; Figure 1).

## Discussion

Sepsis was defined as a systemic inflammatory response to infection. Sepsis and MODS were the most common causes of mortality for septic patients in the ICU [17]. Evidence from both family-based research and genetic association studies demonstrated that a significant portion of the variability in susceptibility to outcome of sepsis was due to genetic factors [18]. Delineating the variation in genes and associated differences in immune inflammatory response might contribute to the development of new genetically tailored diagnostic and therapeutic interventions that will improve outcome in critically ill patients.

RAGE is expressed in many cell types involved in the innate immune system and is able to recognize a wide range of endogenous molecules that are released during various conditions of inflammation and/or injury. Activation of RAGE results in sustained activation of NF-κB, thereby converting transient proinflammatory responses into lasting cellular dysfunction [19]. A number of publications have revealed roles for the -429T/C as a marker for the diabetic/prediabetic state [20], cardiovascular mortality [21] and colorectal cancer [22]. In diabetic subjects, the −429C allele was associated with higher HbA1c levels and occurred with an increased incidence in type 1 diabetic patients [20]. −429 C carriers have an increased risk for colorectal cancer. However, some studies have not revealed many disease associations of -429T/C with type 2 diabetic retinopathy, cardiovascular disease, or incident myocardial infarction [23-25]. Our previous study showed that rs1800625 C allele carriers had lower sepsis morbidity rate and MODS scores [10]. Results from reporter gene assay system suggested that T to C variation of rs1800625 could significantly reduce the transcriptional activity of RAGE promoter. Thus, we enlarged the sample size and added two validation populations to further investigate the clinical relevance of rs1800625 with the development of sepsis and MODS in patients with major trauma.

In both study populations, the −429TT genotype was overrepresented in major trauma patients with sepsis and those with higher MODS scores, suggesting a susceptible role of the TT genotype to the onset of sepsis and MODS. In our study, we further investigated the association of rs1800625 with RAGE protein expression. Consistently, we found that RAGE expression was shown to be well associated with rs1800625 polymorphism, −429TT carriers have higher RGAE protein level. Thus, the rs1800625 polymorphism might be a causal risk allele for sepsis and MODS in patients with major trauma, so it might be used as a biomarker.

However, there are several limitations in our study. First, our study was restricted to Han Chinese and whether the findings can be generalized to other ethnic groups needs further evaluation. Second, the sample size of patients we recruited, though being adequate for the Chongqing cohort, was not enough for the Yunnan and Zhejiang cohorts. The values of power for rs1800625 are shown to be 0.68 and 0.71, respectively, at a significance of 0.05. Additional large studies are needed for the validation cohorts.

## Conclusions

The present study enlarged the sample size and added two validation populations to further investigate the clinical relevance of rs1800625 with the development of sepsis and MODS in patients with major trauma. The −429TT genotype was overrepresented in major trauma patients with sepsis and those with higher MODS scores both in Chongqing and two validation cohorts, suggesting a susceptible role of the TT genotype to the onset of sepsis and MODS. In addition, RAGE expression was shown to be well associated with rs1800625 polymorphism, −429TT carriers have higher RGAE protein level.

## Key messages

- Rs1800625 reveals a strong clinical relevance, showing lower sepsis morbidity rate and MODS scores in the patients with the variant C allele both in the Chongqing, Yunnan and Zhejiang cohorts.
- RAGE expression was also shown to be well associated with rs1800625 polymorphism, −429CC carriers have lower RGAE protein level.

## Abbreviations

ANOVA: analysis of variance
AGEs: advanced glycation end products
CI: confidence interval
CLP: cecal ligation and puncture
DAMPs: damage-associated molecular patterns
FITC: fluorescein isothiocyanate
HMGBl: high-mobility group box-1
HWE: Hardy-Weinberg equilibrium
IgG: immunoglobulin G
ISS: Injury Severity Score
LPS: lipopolysaccharide
MAF: minor allele frequency
MFI: mean fluorescence intensity
MODS: multiple organ dysfunction syndrome
NF-κB: nuclear factor-κB
OR: odds ratio
PAMPs: pathogen-associated molecular patterns
PRR: pattern-recognition receptor
RAGE: the receptor for advanced glycation end products
SNPs: single nucleotide polymorphisms
TNFα: tumor necrosis factor alpha
